# Exploring the Binding Nature of Pyrrolidine Pocket-Dependent Interactions in the Polo-Box Domain of Polo-Like Kinase 1

**DOI:** 10.1371/journal.pone.0080043

**Published:** 2013-11-06

**Authors:** Ravichandran N. Murugan, Mija Ahn, Woo Cheol Lee, Hye-Yeon Kim, Jung Hyun Song, Chaejoon Cheong, Eunha Hwang, Ji-Hyung Seo, Song Yub Shin, Sun Ho Choi, Jung-Eun Park, Jeong Kyu Bang

**Affiliations:** 1 Division of Magnetic Resonance, Korea Basic Science Institute, Ochang, Chung-Buk, Republic of Korea; 2 Department of Bio-Materials, Graduate School and Department of Cellular & Molecular Medicine, School of Medicine, Chosun University, Gwangju, Republic of Korea; 3 Dong-A ST, Research Laboratories, YongIn, Gyeonggi-do, Republic of Korea; 4 Laboratory of Metabolism, Center for Cancer Research, National Cancer Institute, National Institutes of Health, Bethesda, Maryland, United States of America; University of Edinburgh, United Kingdom

## Abstract

**Background:**

Over the years, a great deal of effort has been focused on the design and synthesis of potent, linear peptide inhibitors targeting the polo-like kinase 1 (Plk1), which is critically involved in multiple mitotic processes and has been established as an adverse prognostic marker for tumor patients. Plk1 localizes to its intracellular anchoring sites via its polo-box domain, and inhibiting the Plk1 polo-box domain has been considered as an approach to circumvent the specificity problems associated with inhibiting the conserved adenosine triphosphate-binding pocket. The polo-box domain consists of two different binding regions, such as the unique, broader pyrrolidine-binding pocket and the conserved, narrow, Tyr-rich hydrophobic channel, among the three Plk polo-box domains (Plks 1–3), respectively. Therefore, the studies that provide insights into the binding nature of the unique, broader pyrrolidine-binding pocket might lead to the development of selective Plk1-inhibitory compounds.

**Methodology/Principal Findings:**

In an attempt to retain the monospecificity by targeting the unique, broader pyrrolidine-binding pocket, here, for the first time, a systematic approach was undertaken to examine the structure-activity relationship of *N-*terminal-truncated PLHSpTM derivatives, to apply a site-directed ligand approach using bulky aromatic and non-aromatic systems, and to characterize the binding nature of these analogues using X-ray crystallographic studies. We have identified a new mode of binding interactions, having improved binding affinity and retaining the Plk1 polo-box domain specificity, at the pyrrolidine-binding pocket. Furthermore, our data revealed that the pyrrolidine-binding pocket was very specific to recognize a short and bulky hydrophobic ligand like adamantane, whereas the Tyr-rich hydrophobic channel was specific with lengthy and small hydrophobic groups.

**Conclusion/Significance:**

The progress made using our site-directed ligands validated this approach to specifically direct the ligand into the unique pyrrolidine-binding region, and it extends the applicability of the strategy for discovering selective protein-protein interaction inhibitors.

## Introduction

**Figure 1:**
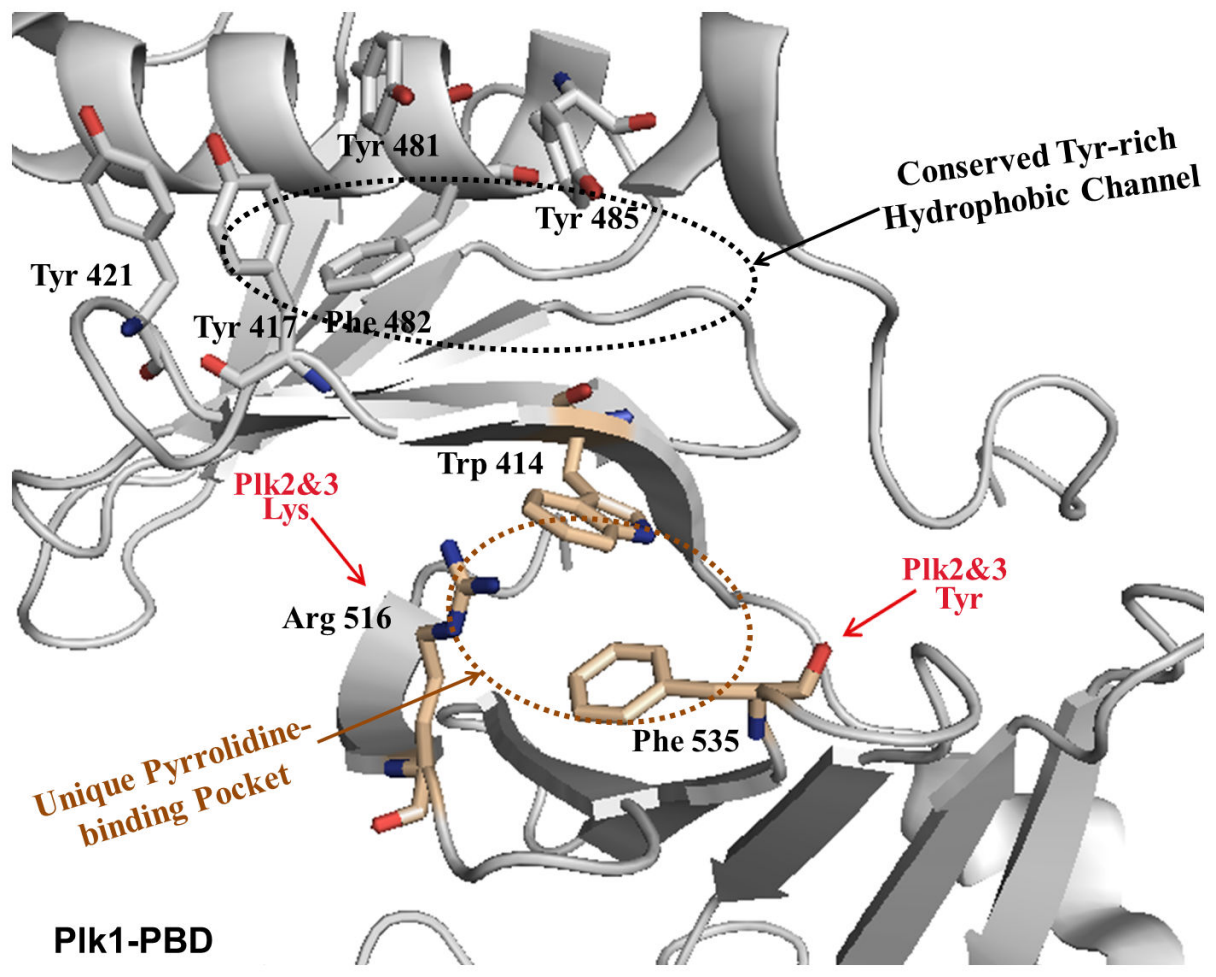
The crystal structure of the previously reported unligated Plk1 PBD, which shows the presence of both the unique pyrrolidine-binding pocket and conserved, Tyr-rich hydrophobic channel.

Intracellular protein-protein interactions play a major role in many signaling pathways, yet they have frequently proven to be a difficult target for small-molecule chemistry, often reflecting a protein interface that is large and discontinuous [1−5]. For instance, polo-like kinase 1 (Plk1, a member of five Plks), a validated anticancer target, has a unique exterior surface (polo-box domain, PBD) composed of charged, hydrophobic, and hydrophilic domains whose phosphorylation-dependent protein-protein interactions are crucial in regulating mitotic progression and cell proliferation. Plk1 is overexpressed in a broad spectrum of human cancers, and elevated Plk1 activity is thought to promote tumorigenesis, while Plk2 and Plk3 play key roles in genetic stability and preventing oncogenic transformation. A large body of evidence suggests that PBD interacts with phosphoserine/phosphothreonine (pS/pT)-containing motifs, and directs the *N-*terminal catalytic activity to specific subcellular localizations. Moreover, targeting PBD could be an alternative strategy used to overcome the cross-reactivity commonly associated with kinase domain inhibitors [6−8]. Extensive studies on the interaction between Plk1 and its centromere/kinetochore-associated binding target, called polo-box-interacting protein 1 (PBIP1) [9], led to the discovery of a minimal peptide, PLHSpT (residues 74–78 of PBIP1). The *N-*terminal Pro residue on PLHSpT is crucial in conferring specificity by docking its side chain into a unique hydrophobic core (hereafter called the pyrrolidine-binding pocket), which is surrounded by the Trp414, Phe535, and Arg516 residues (Figure 1) [10]. As a result, several attempts have been made to generate PLHSpT derivatives, which led to the identification of a narrow, Tyr-rich hydrophobic channel that recognizes its ligands through the hydrophobic interactions of four aromatic residues (Tyr 417, Tyr 421, Tyr 481, and Phe 482) with enhanced anti-Plk1 PBD activity (Figure 1) [11−18]. Since then, the Tyr-rich hydrophobic channel has been targeted to develop the potent Plk1 PBD peptide inhibitors [14−18]. But, the above channel is conserved among the three Plks (Plk1–3) [11], and as a result, a loss of Plk1 PBD monospecificity was observed [12,14,15]. In contrast to the deep understanding of the Tyr-rich hydrophobic channel, the binding nature of the pyrrolidine-binding pocket was not explored much on a molecular basis, even though there has been a high possibility of achieving monospecificity at this binding site. Additionally, the co-crystal structure of Plk1 PBD in complex with the PLHSpT ligand showed that the unique pyrrolidine-binding pocket covers the broader region, suggesting the presence of an unexplored binding surface on PBD [10]. Therefore, here, we would like to specifically exploit the unique pyrrolidine-binding pocket using site-directed ligands that could improve the binding affinity, while retaining Plk1 PBD specificity in comparison to the parent PLHSpT peptide. 

 In this study, a series of PLHSpT-derived peptide analogues, with *N-*terminal Pro-4 acylated by diverse hydrophobic and aromatic functional groups, were prepared and tested to explore the binding nature of the pyrrolidine-binding pocket, in addition to improving Plk1 PBD inhibition. As we report here, during the course of these studies, we observed the unexpected dual-binding ability of the *N-*terminal-truncated peptide derivative, PL-49. Furthermore, the crystallographic analysis of Plk1 PBD, in complex with either PL-42 or PL-55, unambiguously identified a new mode of binding interactions, having improved binding affinity and retaining Plk1 PBD specificity, at the pyrrolidine-binding pocket. Moreover, the investigation of the phosphopeptide-binding pocket using site-directed ligands also revealed that the pyrrolidine hydrophobic pocket was very specific to recognize the short and bulky hydrophobic ligands, such as the adamantane and trimethoxyphenyl moieties, whereas the Tyr-rich hydrophobic channel was specific with lengthy and small hydrophobic functional groups. Our study reveals, for the first time, the structural elements that are crucial in providing steric constraints on parent peptides for the deep understanding of the pyrrolidine-binding region, and that exhibit pyrrolidine pocket-specific, potent Plk1 PBD inhibition. 

## Results and Discussion

### Structure-guided design of *N-*terminal-truncated linear peptides

 To design the site-specific Plk1 PBD peptide inhibitors that might exploit the unique pyrrolidine-binding pocket of Plk1 PBD, first, we closely examined the binding nature of Plk1 PBD in complex with phosphopeptide ligands. In particular, analyzing the crystal structure of Plk1 PBD in complex with PLHSpT (PDB ID: 3HIK) revealed that the N-terminal Pro residue was crucial in conferring specificity by docking its side chain into a hydrophobic core surrounded by the Trp414, Phe535, and Arg516 residues, and by hydrogen bonding its carbonyl group with the Arg516. The specificity appears to stem from the *N-*terminal Pro residue’s failure to interact with the PBDs of Plk2 and Plk3, which possess the Lys and Tyr residues at positions corresponding to the Arg516 and Phe535 residues, respectively, in Plk1 PBD. Moreover, the addition of *C-*terminal Ala and the deletion of *N-*terminal Pro in PLHSpT (LHSpTA) bound to Plk1 PBD almost as efficiently as the PLHSpT did [10]. The results described above suggest that adding the *C-*terminal hydrophobic residue and substituting the bulky, pi-pi-mediating functional groups at the broader pyrrolidine-binding region of PBD, which is surrounded by Trp414 and Phe535, could provide potent, site-specific Plk1 PBD inhibition. On the other hand, the PBD residues of the narrow, Tyr-rich hydrophobic channel are conserved in the PBDs of closely related family members Plk2 and Plk3, and the ligands that participate in this hydrophobic channel showed cross-reactivity with Plk2 PBD compared to the monospecific, PLHSpT peptide [12,14,15].

**Figure 2:**
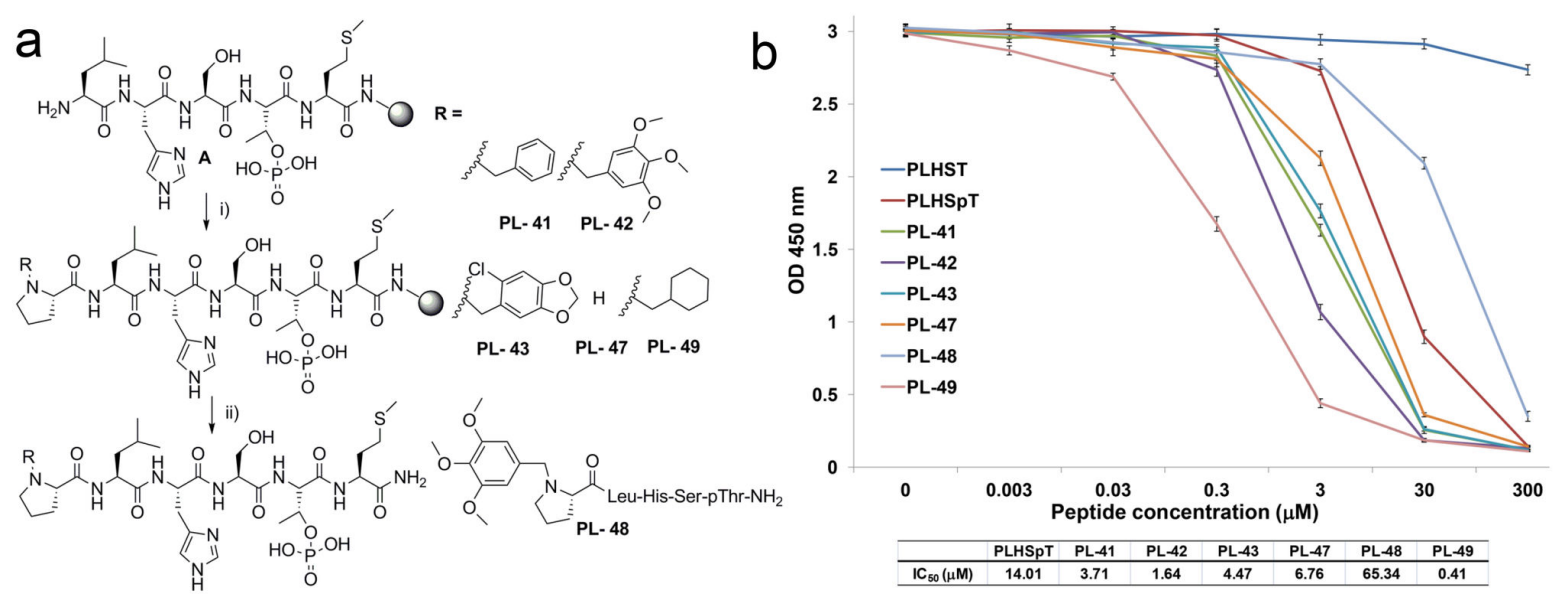
First-phase diversification of peptides, and quantification of their inhibitory activities against Plk1 PBD. (A) First-phase *N-*terminal-truncated PLHSpT derivatives generated using solid-phase peptide synthesis (SPPS). i) RCH_2_-Pro-OH, HBTU, HOBt, DIPEA, DMF, 4 h; ii) TFA/TIS/H_2_O (90:5:5), 2.5 h; (b) ELISA-based Plk1 PBD-binding assay result using first-phase *N-*terminal-truncated peptide derivatives. Representative graphs from three independent experiments are shown (O.D., optical density).

 By considering the views described above, we designed the first-phase, N-terminal-truncated peptides to investigate the effect of *N*- and C-terminal modifications in recognizing the broader pyrrolidine-binding region. In order to mediate pi-pi-stacking interactions, as well as additional hydrophobic interactions, aromatic moieties and the Met residue were incorporated at the *N*- and C-terminus of the PLHSpT parent peptide. This was accomplished by acylating the Leu-3 residue of LHSpTM precursors using *N*-alkylated Pro derivatives in the presence of 1-*O*-Benzotriazole-*N*,*N*,*N*′,N′-tetramethyluronium hexafluoro-phosphate (HBTU), 1--hydroxybenzotriazole (HOBt), and N,N-diisopropylethylamine (DIEA) as coupling agents (Figure 2A). All the linear precursors were prepared with the standard fluorenylmethoxycarbonyl (Fmoc)-based, solid-phase peptide synthesis using Rink amide resin. The binding affinities of the above *N-*terminal-truncated PLHSpTM derivatives were evaluated using an enzyme-linked immunosorbent assay (ELISA)-based Plk1 PBD-binding assay (Figure 2B). We observed that the simple Met addition at the C-terminus of linear PLHSpT increased the binding affinity two-fold (PL-47, Figure 2); hence, Met was added at the C-terminus for later modifications. As expected, incorporating aromatic functional groups (PL-41, PL-42, and PL-43) substantially improved Plk1 PBD inhibitory activity. Among the pi-pi mediating aromatic functional groups, the bulky trimethoxy-substituted phenylmethyl (benzyl) group on PL-42 ((MeO)_3_PhCH_2_PLHSpTM, IC_50_ = 1.48 µM, Figure 2) gave higher binding affinity than either the unsubstituted phenylmethyl group (PL-41) or chloro-substituted, fused phenylheterocyclic group (PL-43). To further elucidate the importance of C-terminus Met on PL-42, we synthesized PL-48 lacking the Met residue at C-terminus and tested Plk1 PBD-binding affinity. As expected, deleting C-terminus Met on PL-42 (PL-48) has dramatically decreased the binding affinity by 40 (Figure 2). 

 Another important finding was that the PL-49 (cyclohexylmethyl-PLHSpTM) with a cyclohexyl moiety at the N-terminus was synthesized as a negative control against PL-41. But, the binding assay showed the unexpected higher potency of PL-49 (IC_50_ = 0.36 µM) over PL-42 (IC_50_ = 1.48 µM), even though it was not able to mediate the pi-pi-stacking interactions (Figure 2). In order to verify our site-directed ligand approach through pi-pi-stacking interactions with Trp414 and Phe535, and at the same time rationalize the difference in the binding affinity between PL-49 and PL-42, crystallographic studies were carried out with Plk1 PBD in complex with phosphopeptide ligands (PL-42 and PL-49). 

**Figure 3:**
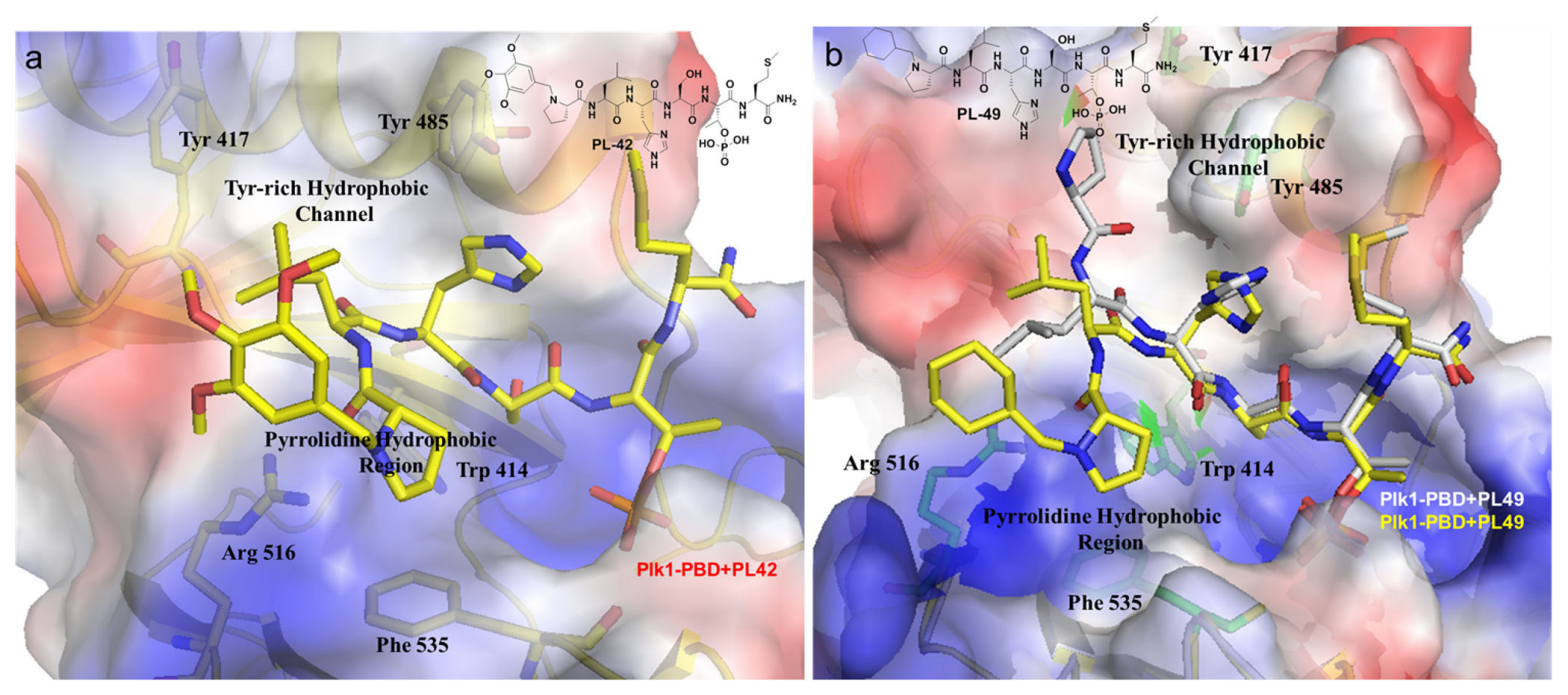
Crystal structures of Plk1 PBD in complex with PL-42 or PL-49. (A) The crystal structure of the Plk1 PBD-PL-42 complex showed that the *N-*terminal moiety of PL-42 interacts with the pyrrolidine hydrophobic region. (B) The overlay of the two distinct crystal structures of the Plk1 PBD-PL-49 complex revealed that the *N-*terminal cyclohexyl moiety of PL-49 possesses the dual-binding ability and interacts with either the Tyr-rich hydrophobic channel or the pyrrolidine hydrophobic region. Note that since the *N-*terminal arm of PL-49 (gray backbone) that points to the Tyr-rich hydrophobic channel could not be localized in the density maps because of its high flexibility, we were not able to model the cyclohexylmethyl segment using PyMOL [19].

 Surprisingly, the crystal structure of Plk1 PBD in complex with PL-42 showed that the bulky N-terminal trimethoxy-substituted phenylmethyl fragment of PL-42 bound to the broader pyrrolidine-binding pocket instead of mediating a pi-pi-stacking interaction with the surrounding PBD residues (Figure 3A and Table 1). Moreover, the crystal structure also indicated that there were no observable interactions between these *N*- and C-terminus additions and the PBD residues, suggesting that the conformational requirements imposed by the *N-*terminal trimethoxy-substituted benzyl moiety and the C-terminus Met side chain make a significant contribution to the binding. 

**Table 1:** Data collection and refinement statistics.

	PL-42	PL-49	PL-55	PL-74
Data collection				
Space group	*P*2_1_	*P*4_3_2_1_2	*P*2_1_	*C*222_1_
Resolution (Å)	56.30 - 2.0 (2.11 - 2.00)	88.28 - 2.65 (2.74 - 2.65)	50 - 1.58 (1.64 - 1.58)	50 - 2.0 (2.07 - 2.0)
*R*-merge^*^	0.047 (0.249)	0.060 (0.348)	0.051 (0.476)	0.073 (0.346)
reflections	39,962 (5,663)	127,805 (18,563)	104,942	216,609
unique reflections	13,200 (1,912)	32,192 (4,684)	28,953	33,797
redundancy	3.0 (3.0)	4.0 (4.0)	3.6 (3.4)	6.4 (6.1)
completeness (%)	98.1 (97.8)	98.6 (82.8)	99.5 (98.3)	99.8 (99.0)
refinement				
*R*-factor^**^	0.199	0.260	0.230 (0.269)	0.202 (0.221)
free-*R*	0.247	0.259	0.259 (0.289)	0.236 (0.249)
RMSD				
bonds (Å)	0.006	0.014	0.0048	0.0057
angles (°)	1.24	2.03	1.19	1.32
*B*-factors (Å^2^)				
protein	37.5	65.9	22.6	26.6
water	41.6	48.4	27.3	32.0
ligand	35.8	53.6	20.4	30.0

Values in parentheses correspond to the highest-resolution bin

* *R*-sym = ∑ | *I* - <*I*> |/ ∑ *I*

** *R*-factor = ∑| |F_obs_(*hkl*)| |F_calc_(*hkl*)| |/∑ |F_obs_(*hkl*)|

On the other hand, the crystal structure of PL-49, for the first time, revealed the two different binding modes in which the cyclohexyl moiety of PL-49 bound to either the Tyr-rich hydrophobic channel or the pyrrolidine-binding region of Plk1 PBD, due to the crystal structure’s ability to assume dynamic conformational ensembles (Figure 3B and Table 1). Although the X-ray structure did not show any interactions between the *N-*terminal cyclohexyl moiety and the surrounding PBD residues, the observed higher binding affinity of PL-49 (IC_50_ = 0.36 µM) than that of PL-42 is probably due to the strong hydrophobic interactions from the dual-binding modes. The results described above clearly demonstrated that the aromatic group at the Pro-4 N-terminus was not essential to improving the binding affinity because the expected pi-pi-stacking interactions were not seen from the crystal structure of PL-42. Instead, the bulky hydrophobic interaction-mediating groups similar to the trimethoxy-substituted ring systems at the N-terminus of PL-42 could be the right candidates to accommodate the broader pyrrolidine-binding region and improve the binding affinity. Needless to say, the additional Met residue located at the C-terminus of PL-42 also played a crucial role in improving Plk1 PBD-binding affinity. 

**Figure 4:**
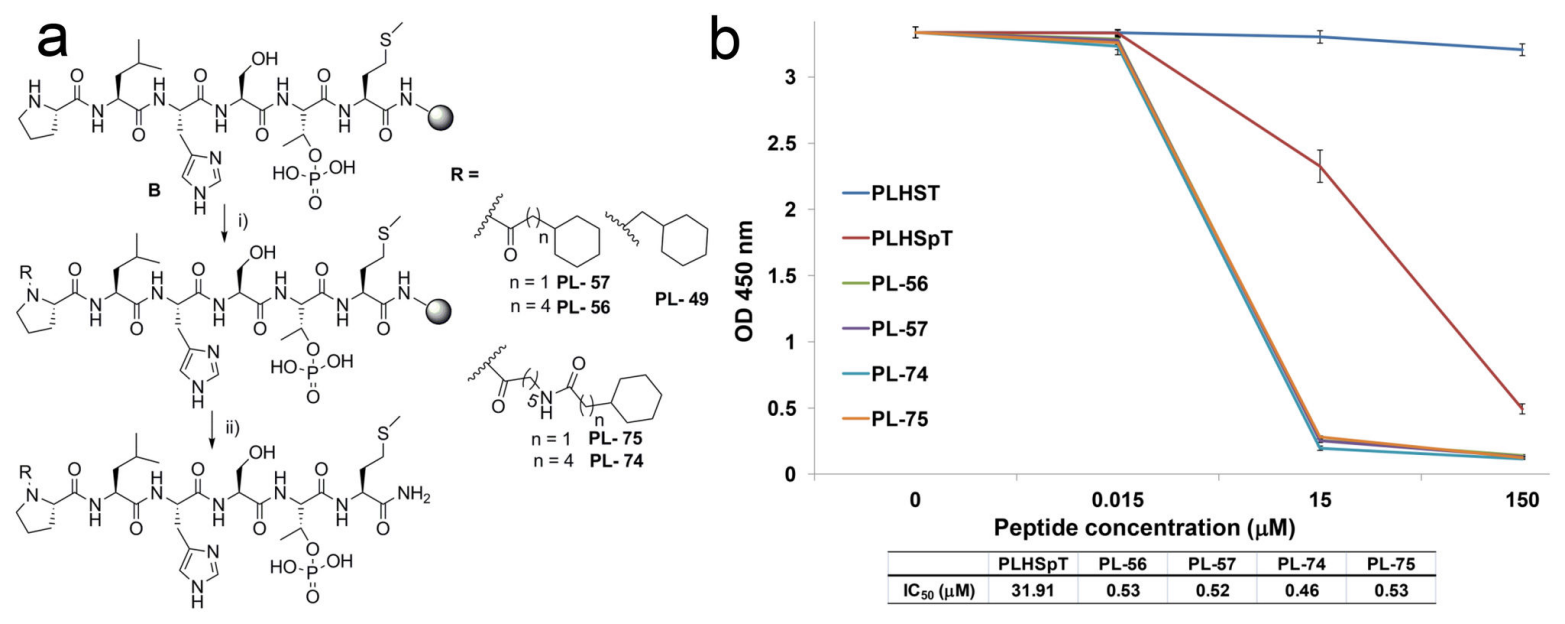
First-phase diversification of peptides, and quantification of their inhibitory activities against Plk1 PBD. (A) First-phase *N-*terminal-truncated peptides generated using solid-phase peptide synthesis (SPPS). i) R-COOH, HBTU, HOBt, DIPEA, DMF, 2 h; ii) TFA/TIS/H_2_O (90:5:5), 2.5 h; (B) ELISA-based Plk1 PBD-binding assay result using first-phase *N-*terminal-truncated peptide derivatives. Representative graphs from three independent experiments are shown (O.D., optical density).

Consistent with the Tyr-rich binding mode of PL-49, it was recently reported that replacing the Pro-4 position on the PLHSpT peptide using alkylated phenyl substituent also bound preferentially to the narrow, Tyr-rich hydrophobic channel [14,15]. Therefore, we have generated various N-terminal cyclohexyl-truncated analogues (PL-56, -57, -74, and -75) to study the binding nature of PL-49 with respect to the variation in chain lengths on Plk1 PBD-binding regions (Figure 4). Accordingly, we have synthesized the analogues by the direct acylation of the Pro-4 residue of linear precursors with commercially available acid derivatives; thus, the simplified procedure avoided the solution-phase synthesis of precursors for the *N-*terminal Pro-4 modification (Figure 4A). 

 However, the binding affinity of these analogues displayed equal potency (PL-56, -57, -75, and -74; Figure 4B) when alkyl tethers were increased from one to eleven by atoms on PL-49. Moreover, in contrast to the previous reports in which the longer alkylphenyl group increased the binding affinity [12,14,15], the N-terminus cyclohexyl methyl moiety on PL-57 was strong enough to attain the observed Plk1 PBD-binding affinity in comparison to PL-74 (Figure 4B). 

**Figure 5:**
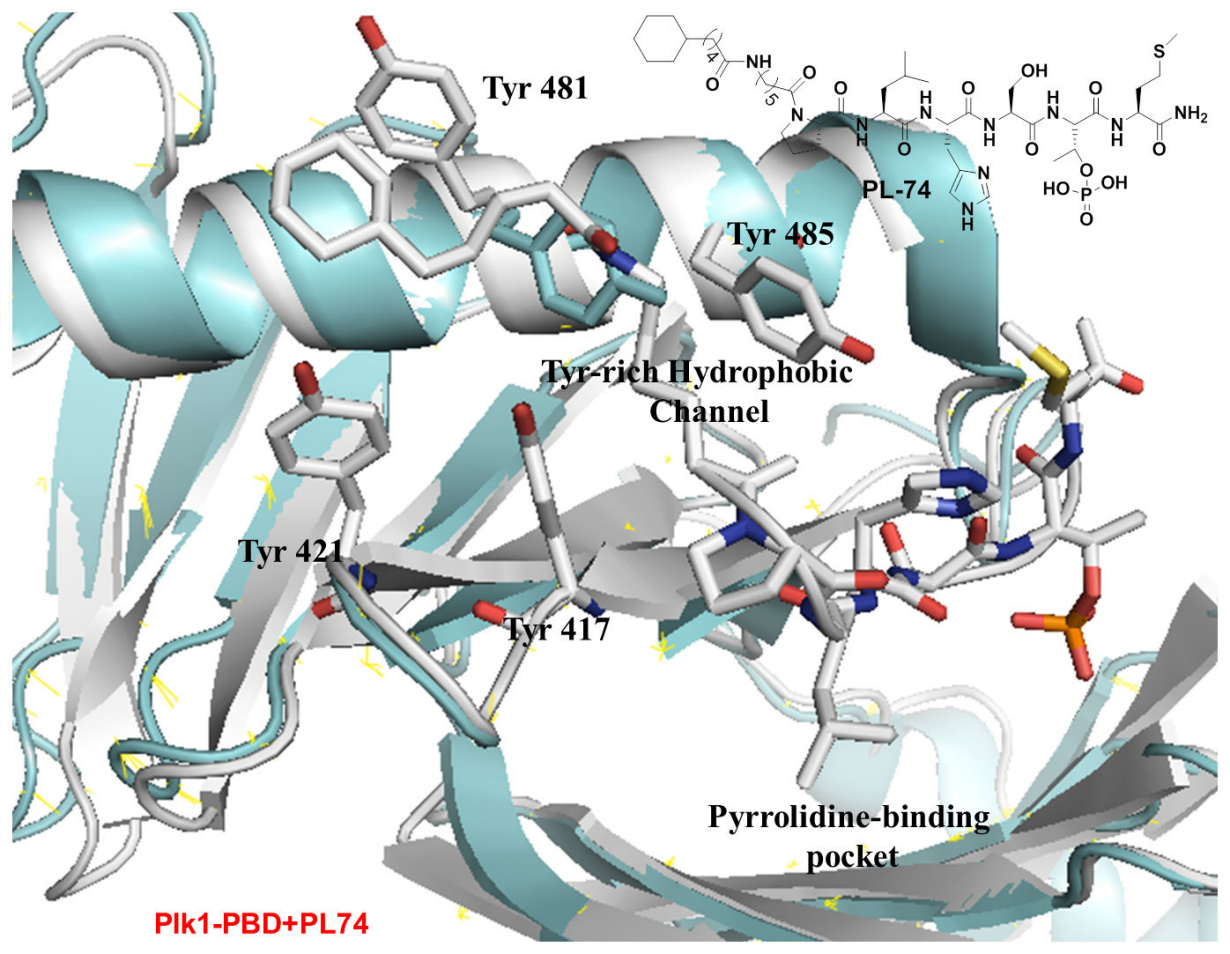
Crystal structures of Plk1 PBD in complex with PL-74. The overlay of the crystal structures of the Plk1 PBD-bound PL-74 (white) over the previously reported unligated Plk1 PBD (cyan) revealed that the *N-*terminal cyclohexyl moiety of PL-74 bound deeply into the Tyr-rich hydrophobic channel.

To determine the binding nature of PL-74, we solved the X-ray co-crystal structure of PL-74 in complex with Plk1 PBD. As expected, when the cyclohexyl moiety at the *N-*terminal of PL-49 was extended, the resulting PL-74 was directed to the Tyr-rich hydrophobic channel and lost the dual-binding ability. Similar to the previously reported peptide derivatives that were bound to the Tyr-rich hydrophobic channel [12,14,15], the side chain of Tyr481 rotates away from the domain to open the channel. But, in contrast, the position of Tyr481 was parallel to the cyclohexyl moiety because of the closer and deeper hydrophobic binding interactions from the *N-*terminal substitution on PLHSpTM (Figure 5 and Table 1). 

### Second-phase, *N-*terminal-truncated peptide derivatization

**Figure 6:**
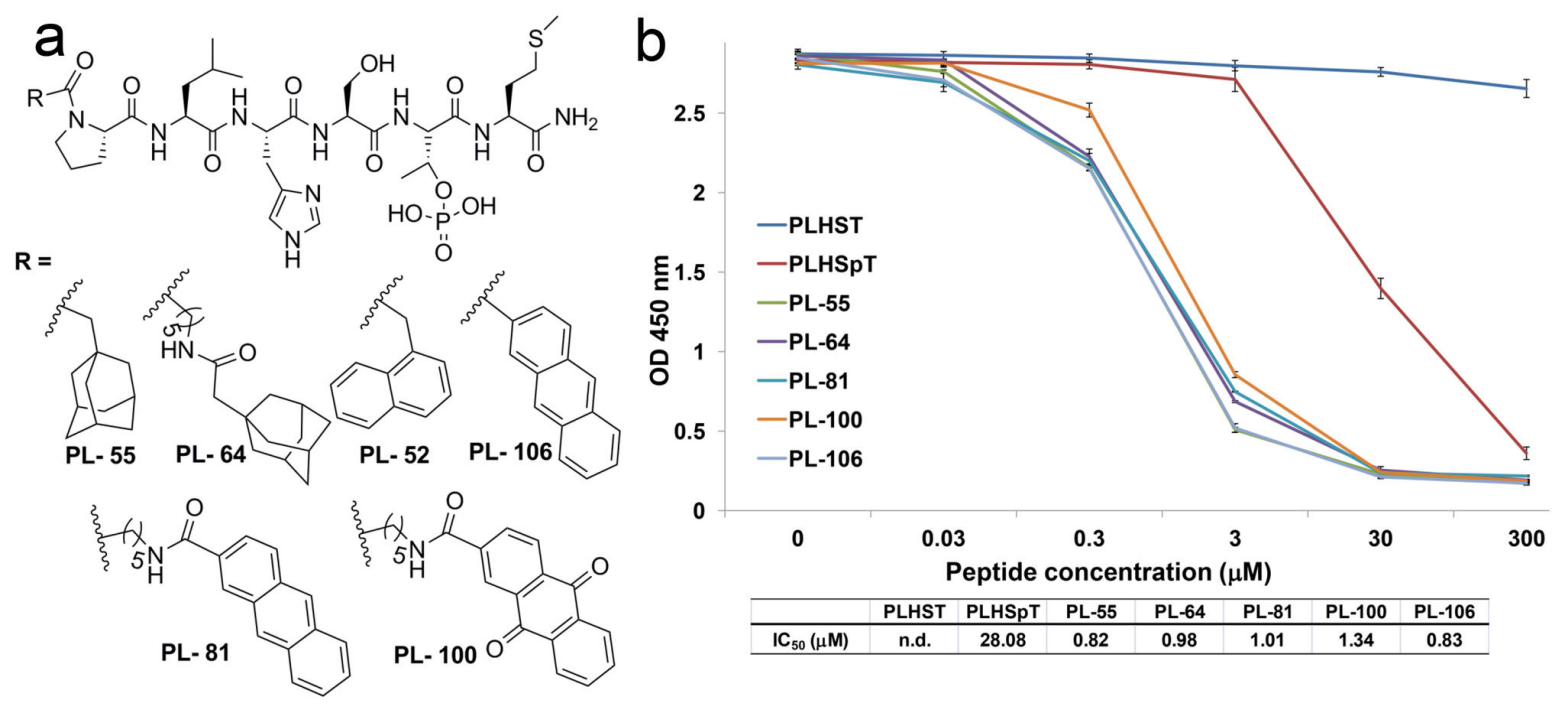
Selected *N-*terminal peptide derivatives synthesized from the second-phase diversification and quantification of their inhibitory activities against Plk1 PBD. (A) Second-phase N-terminal-truncated PLHSpTM peptide derivatives generated using solid-phase peptide synthesis (SPPS) to study the binding nature of the pyrrolidine-binding region. (B) ELISA-based Plk1 PBD-binding IC_50_ graph (O.D., optical density). A representative graph from three independent experiments.

 Based on our first screening of peptide derivatives, we found that the bulky, uncharged hydrophobic fragments at the N-terminus of PLHSpTM could enhance Plk1 PBD inhibition by occupying the broader binding pocket at the unique, pyrrolidine-binding region. Accordingly, we expanded the series of *N-*terminal-truncated PLHSpTM derivatives using diverse, bulky, short, and long aromatic and non-aromatic ring systems, which seem to afford an ideal opportunity to cover the broader binding pocket for the specific recognition of the pyrrolidine-binding region (Figure 6A). 

 Our assay results revealed that the peptide-containing adamantyl group at the N-terminus (PL-55) showed one of the highest binding affinities, compared to PL-42. On the other hand, the peptide derivatives in the presence or absence of the alkyl chain tether on either adamantyl (PL-64) or di- and tri-cyclic aromatic systems (PL-52, -81, -100, and -106) showed a similar binding affinity, compared to PL-55 (Figure 6B). All the results described above supported our hypothesis that the pyrrolidine-binding pocket was broader to accommodate the bulky groups and mediate the hydrophobic interactions rather than the pi-pi-stacking interactions. 

**Figure 7:**
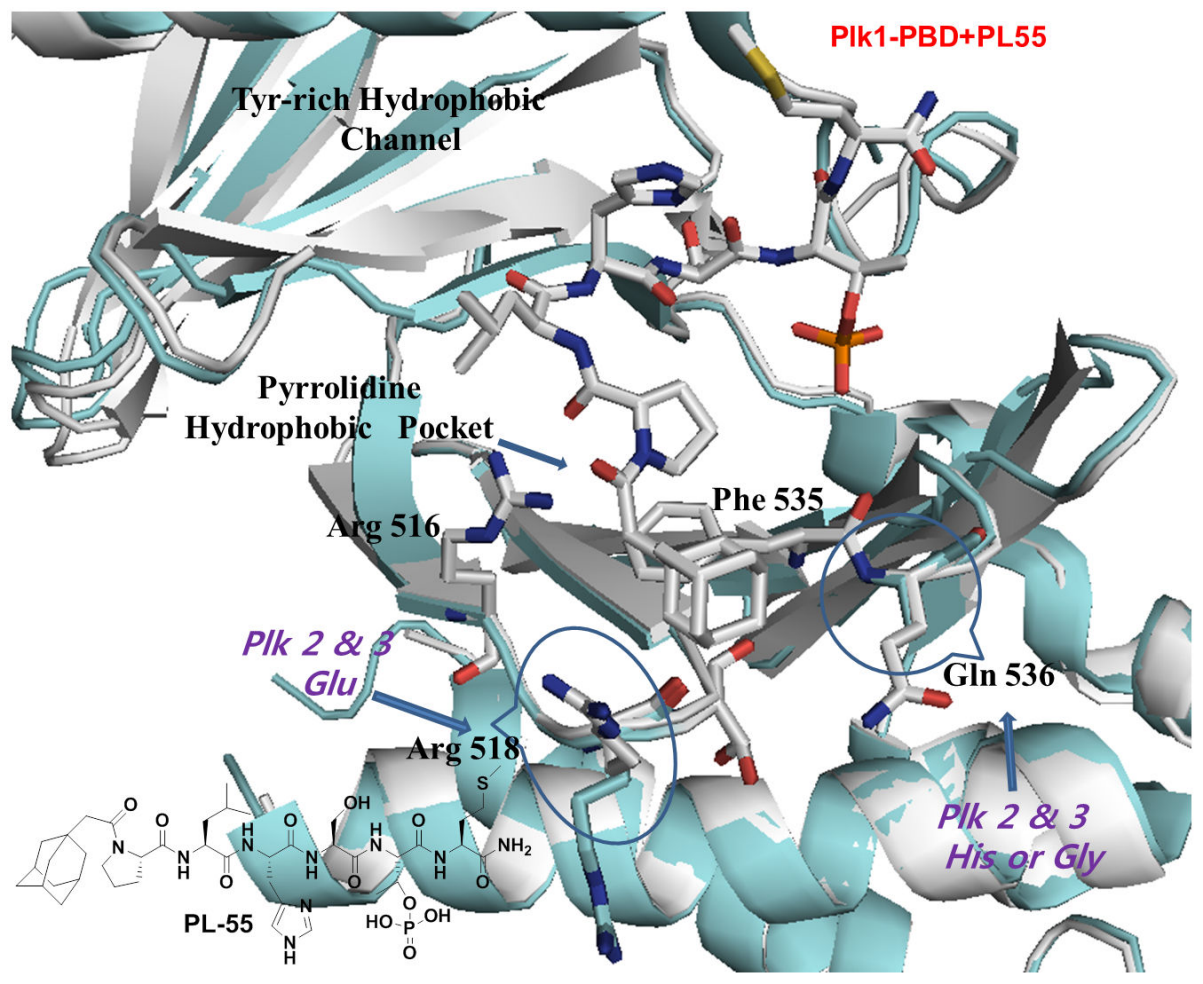
Crystal structures of Plk1 PBD in complex with PL-55. The overlay of the two distinct crystal structures of Plk1 PBD in complex with either PL-55 (gray backbone) or unligated Plk1 PBD (cyan) revealed that the *N-*terminal adamantyl moiety of PL-55 buried into the broader pyrrolidine hydrophobic region and interacted with unprecedented PBD residues such as Arg 518 and Gln 536.

 Finally, in an attempt to determine the molecular basis for the enhanced binding affinity on PL-55, we solved the co-crystal structure of Plk1 PBD in complex with PL-55. More importantly, the crystal structure of Plk1 PBD in complex with PL-55 further reinforces our detailed understanding of the unique binding nature of the pyrrolidine-binding region, showing that the hydrophobic interactions between the adamantyl moiety and the unprecedented, new PBD-interacting elements, such as Arg518 and Gln536, played an important role for the specific tight binding into the broad, pyrrolidine hydrophobic pocket. Notably, the side-chain conformation of Arg518 rotated by around 120 degrees to accommodate the hydrophobic adamantyl moiety presented to the pocket. Furthermore, the above two PBD residues were either not identified in the previous crystal studies due to the high flexibility, or they reside in the reverse direction of the peptide chain [10,12]. Thus, the crystal data indicated that the bulky adamantyl substitution in PL-55 acted as a site-directed moiety that prevented the *N-*terminal Pro motif from turning into the narrow passage of the Tyr-rich hydrophobic channel (Figure 7 and Table 1). Moreover, in contrast to the dual-binding ability of PL-49, PL-55 could not provide the additional hydrophobic interactions from the Tyr-rich hydrophobic channel; thus, PL-55 showed a small decrease in potency (Figures 2 and 6).

 All the results described above suggest that the site-directed ligand approach by means of introducing bulky functional moieties on the parent peptide can be successfully applied to specifically target the unique, broader pyrrolidine-binding pocket. 

### Impact on selectivity

**Figure 8:**
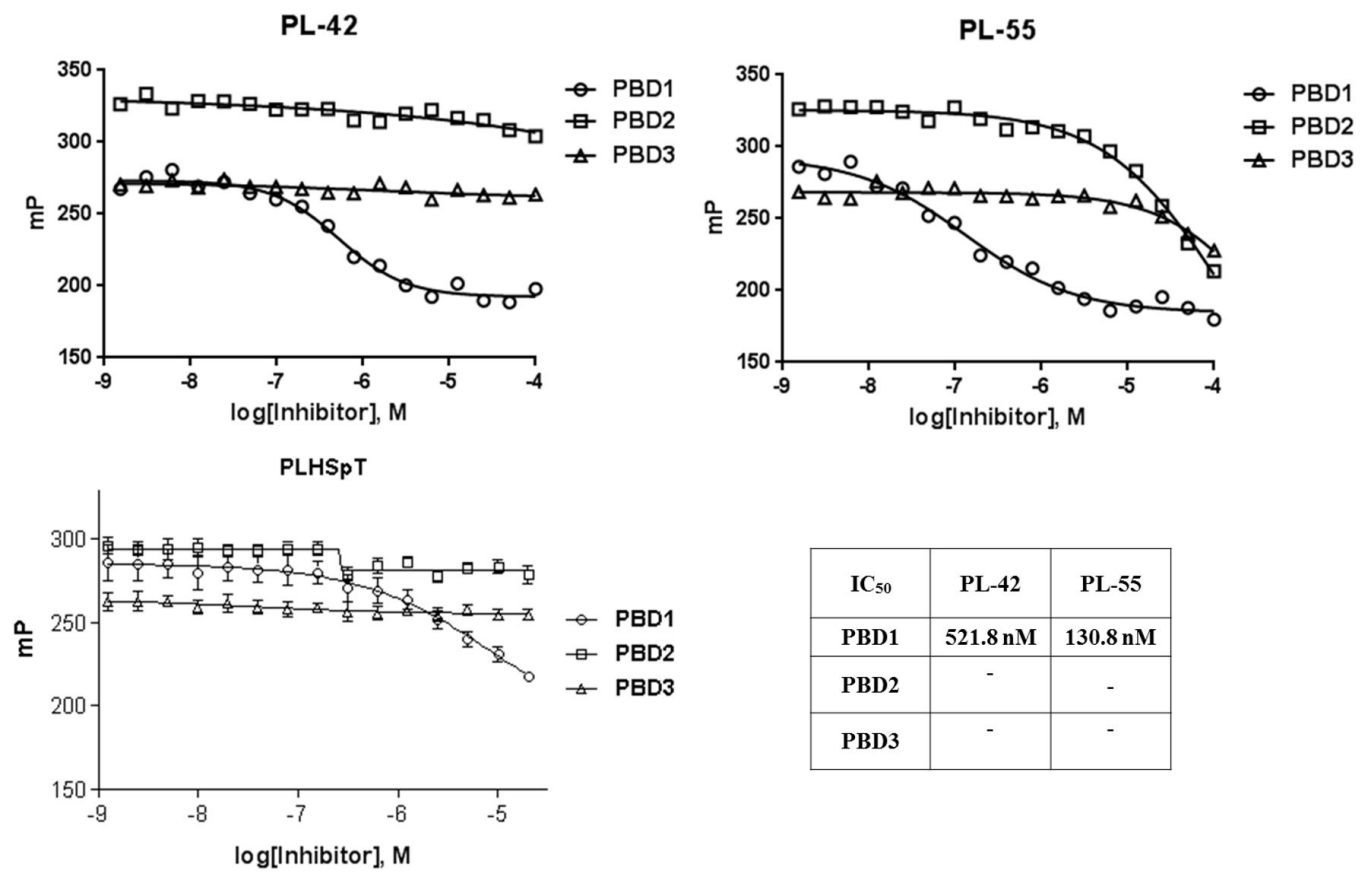
Comparison of fluorescence polarization–based assays showing the selective inhibition of Plk1 PBD over Plk2 and 3 PBDs by PL-42 and PL-55.

 To examine the binding specificity of PL-42 and PL-55 in comparison to that of parent PLHSpT, we carried out a specificity test against PBDs from closely related Plk1, 2, and 3. The Plk4 PBD was not included because of the distinct binding nature of this protein. Site-directed peptide inhibitors are thought to increase potency by lowering the entropic barrier to complex formation. This approach could also potentially enhance the specificity of the inhibitor by limiting its interaction modes with the target protein [20]. The assay results showed that both of these *N-*terminal-truncated peptide derivatives exhibited monospecificity against Plk1 PBD (Figure 8), even though their binding affinities were approximately 10- (PL-42) to 36 (PL-55)-fold higher than parent PLHSpT. The observed monospecificity is presumably due to the specific recognition of bulky, hydrophobic groups substituted at the *N-*terminal PLHSpTM peptide derivative by the unique, broader pyrrolidine-binding pocket. 

## Conclusion

In this study, we sought to use the effect of steric hindrance from the bulky *N-*terminal truncations to identify ligands that could specifically target the pyrrolidine-binding pocket rather than the conserved, Tyr-rich hydrophobic channel. As expected, PL-42 and PL-55, having bulky *N-*terminal substituents like trimethoxy-substituted benzyl or the adamantyl group, buried into the broader pyrrolidine-binding pocket, which was confirmed using X-ray crystallography, and has been shown to participate with the newly identified unique PBD residues that mediate hydrophobic interactions. Given the ever-increasing number of peptide-derived Plk1 inhibitors, we believe that the application of site-directed peptide inhibitors against Plk1 has the potential to provide further valuable insights into molecular recognition by the phosphopeptide-binding pockets. These observations demonstrated a degree of plasticity within the pyrrolidine-binding pocket that enables it to accommodate bulky, *N-*terminal-truncated peptide inhibitors rather than the narrow, Tyr-rich hydrophobic channel. Our results provide additional support that the pyrrolidine-binding pocket is a unique region, even among the Plks, through the identification of new PBD-interacting residues, such as Arg 518 and Gln 536, which are not conserved in the PBDs of the closely related family members Plk2 and Plk3. Moreover, the insights gained from the binding nature of the unique pyrrolidine-binding region might be used successfully for developing clinically valuable monospecific anti-Plk1 inhibitors.

## Materials and Methods

 All reactions requiring anhydrous conditions were conducted in flame-dried reaction vessels under a positive pressure of Ar. Reagents were obtained commercially (Sigma-Aldrich or TCI) and used without further purification. For the chromatography analysis, high-performance liquid chromatography (HPLC)-grade solvents such as hexane, ethyl acetate, methylene chloride, and methanol were used. Thin layer chromatography (TLC) was performed on analytical Merck silica gel 60 F254 and visualized under ultraviolet light first, then by warming in the presence of ninhydrin solution (approx. 0.1% w/v in ethanol). Flash chromatography was performed on Merck silica gel 60 (230–400 mesh). NMR spectra were recorded on a Bruker Avance 300 spectrometer at 300 for ^1^H with CDCl_3_. Chemical shifts (δ) were reported in parts per million (ppm), measured relative to an internal standard, and the coupling constants (*J*) are expressed in hertz (Hz). The molecular masses of purified peptides were determined using matrix-assisted laser-desorption ionization time-of-flight mass spectrometry (MALDI-TOF MS) (Shimadzu, Japan). All organic extracts were dried using magnesium sulfate and concentrated under reduced pressure by rotary evaporation. Reverse-phase HPLC analysis (RP-HPLC) was carried out at 230 nm on an Agilent HPLC system equipped with a C_18_ analytical column (250 × 10mm, 5 micron). Two different linear gradients of 0.05% aq. TFA (eluent A) and 0.05% TFA in CH_3_CN (eluent B) were used with a flow rate of 2.5 mL/min at 25 °C. 

### General procedures for peptide synthesis

All peptides were prepared using the standard Fmoc-based, solid-phase peptide synthesis (SPPS) method using Rink amide resin, with an initial loading of 0.61 mmol/g, unless otherwise noted. Fmoc-Thr(PO(OBzl)OH)-OH and other Fmoc(fluorenylmethoxycarbonyl)-protected amino acids were purchased from Novabiochem. Resins were swollen in *N*,*N-*dimethylformamide (DMF) for 45 min prior to synthesis. For sequence extension, the Fmoc-protected amino acid (5.0 eq.) was activated by treatment with HBTU (5.0 eq.) and HOBt (5.0 eq.) in DMF (2 mL) for 2 min. This solution was added to the free amine on resin, along with N,N-diisopropylethylamine (10.0 eq.), and the coupling reaction was allowed to proceed for 1 h with vortex stirring. After washing with DMF, Fmoc de-protection was achieved with 20% piperidine in DMF (1 × 10 min, 2 × 3 min). The resin was washed once again, and the process was repeated for the next amino acid. Finally, the resin was washed sequentially with DMF, methanol, dichloromethane, and ether, and then dried under a vacuum.

### Analytical HPLC conditions

 The peptides were purified to a minimum purity of 97% using a phenomenex column (C_18,_ 250 × 10 mm, 5 micron), with a linear gradient from 5% aqueous acetonitrile (0.05% trifluoroacetic acid) to 95% acetonitrile (0.05% trifluoroacetic acid), over 30 min at a flow rate of 2.5 mL/min and detection at 230 nm. 

### Synthesis of *N-*terminal-truncated peptides

**Figure 9:**
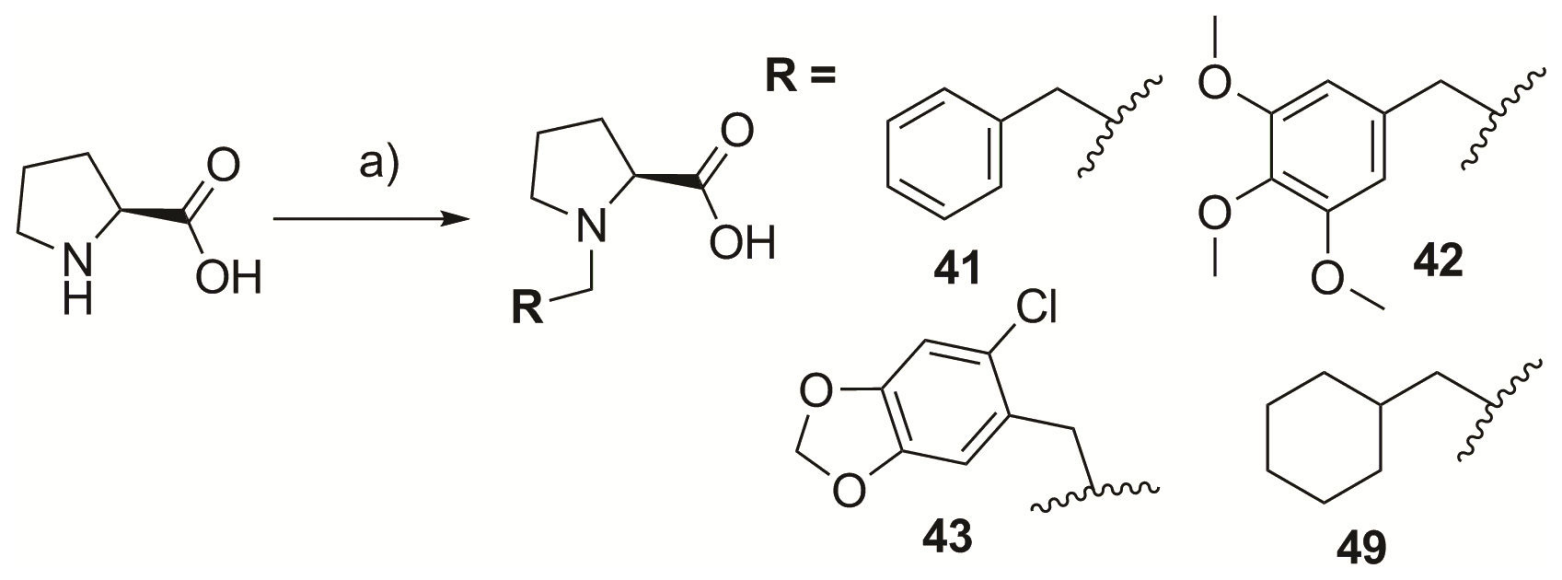
Synthesis of *N-*substituted proline derivatives. Reagents and conditions: (A) R-X, NaOMe/MeOH, 50°C, 12 h.

 To diversify *N-*terminal-truncated peptides, initially, the standard Fmoc-based SPPS was followed to provide rink amide resin-bound peptide, **A** (Figure 2A). Next, acylation of Leu-3 was carried out using the corresponding *N-*substituted proline derivatives. Finally, peptides were cleaved by TFA at room temperature for 2 h, followed by filtration, precipitation in cold ether, centrifugation, and drying under a vacuum (Figure 2A). Direct acylation on *N-*terminal Pro was also performed on the resin-bound PLHSpTM peptide, **B** (Figure 4B), using the acid derivatives (52, 55, 56, 57, 100, and 106) that were purchased from Sigma-Aldrich or TCI. The sequence and structure of each peptide were characterized by mass spectrometry, and the purity of the peptides (>97%) was determined by analytical RP-HPLC. Compounds 41, 42, 43, and 49 were prepared according to the indicated literature procedure (Figure 9) [21].

#### General procedure for preparing (S)-*N*-substituted proline

The synthesis was performed in accordance to Ueki et al [21]. 

#### Synthesis of (S)-*N-*benzyl proline, 41

CH_3_ONa (1.08 g, 20 mmol) and benzyl chloride (1.16 mL, 10.1 mmol) were added slowly to a solution of proline (1.15 g, 10.0 mmol) in methanol (10 mL) under argon atmosphere at room temperature, and allowed to stir overnight at 50 °C (Figure 9). The reaction mixture was diluted with CHCl_3_ and then neutralized with concentrated HCl. The precipitate was filtered using CHCl_3_, and the remaining filtrate was evaporated and slowly precipitated at 5–10 °C using cold acetone solution. The combined precipitates were dried to afford 1.26 g of 41 (61%).

^1^H NMR (300 MHz, CDCl_3_): δ 1.89–2.08 (m, 2H), 2.09–2.30 (m, 2H), 2.82 (m, 1H), 3.58 (m, 1H), 3.76 (t, *J* = 7.2 Hz, 1H), 4.16, 4.30 (ABq, *J* = 12.9, 2H), 7.18–7.48 (m, 5H).

All the values are in agreement with the reported values [21].

#### Linear Peptide, PL-41

Synthesis was carried out on a 0.062 mmol scale. The purity of the crude product was 79% (C_18_ RP-HPLC), and the yield after purification was 15.4 mg (25%, 98% purity) of white powder. RP-HPLC analysis, R_t_ = 13.3 (5–95% B in 30 min). MS (MALDI-TOF) m/z = 854.15 [M+H]^+^.

#### Synthesis of (S)-*N*-3,4,5-trimethoxybenzylproline, 42

Compound 42 was synthesized using the general procedure that was followed for the synthesis of 41 (Figure 9), proline (575 mg, 5 mmol), MeOH (5 mL), CH_3_ONa (540 mg, 10 mmol), and 3,4,5-trimethoxybenzyl chloride (1.01 g, 5.05 mmol). The combined precipitates were dried to afford 500 mg of 42 (34%).

^1^H NMR (300 MHz, CD_3_OD): δ 1.83–2.18 (m, 3H), 2.43 (m, 1H), 3.24 (m, 1H), 3.59 (m, 1H), 3.72 (s, 3H), 3.82 (s, 6H), 3.93 (m, 1H), 4.24, 4.35 (ABq, *J* = 12.8, 2H), 6.84 (s, 2H).

#### Linear Peptide, PL-42

Synthesis was carried out on a 0.061 mmol scale. The purity of the crude product was 74% (C_18_ RP-HPLC), and the yield after purification was 13.4 mg (23%, >98% purity) of white powder. RP-HPLC analysis, R_t_ = 12.0 (5–95% B in 30 min). MS (MALDI-TOF) m/z = 943.1 [M]^+^.

#### Synthesis of (S)-*N*-6-chloro piperonyl proline, 43

Compound 42 was synthesized using the general procedure that was followed for the synthesis of 41 (Figure 9), proline (115.1 mg, 1.0 mmol), MeOH (1 mL), CH_3_ONa (108 mg, 2.0 mmol), and 6-chloro piperonyl chloride (207 mg, 1.0 mmol). The combined precipitates were dried to afford 184 mg of 43 (65%),

^1^H NMR (300 MHz, CD_3_OD): δ 1.84–2.24 (m, 3H), 2.48 (m, 1H), 3.29 (m, 1H), 3.63 (m, 1H), 3.97 (m, 1H), 4.42, 4.53 (ABq, *J* = 13.2, 2H), 6.05 (s, 2H), 6.97 (s, 1H), 7.21 (s, 1H).

#### Linear Peptide, PL-43

Synthesis was carried out on a 0.061 mmol scale. The purity of the crude product was 74% (C_18_ RP-HPLC), and the yield after purification was 13.2 mg (23%, 98% purity) of white powder. RP-HPLC analysis, R_t_ = 12.7 (5–95% B in 30 min). MS (MALDI-TOF) m/z = 931.41 [M]^+^.

#### Synthesis of (S)-*N*-cyclohexylmethyl proline, 49

Compound 49 was synthesized using the general procedure that was followed for the synthesis of 41 (Figure 9), proline (288 mg, 2.5 mmol), MeOH (2.5 mL), CH_3_ONa (270 mg, 5 mmol), and cyclohexylmethyl bromide (351 μL, 2.5 mmol). The combined precipitates were dried to afford 245 mg of 49 (46%).

^1^H NMR (300 MHz, CDCl_3_): δ 0.85–1.30 (m, 5H), 1.54–1.85 (m, 5H), 1.86–2.05 (m, 3H), 2.14 (m, 1H), 2.32 (m, 1H), 2.78–2.97 (m, 2H), 3.03 (m, 1H), 3.82 (m, 1H), 3.93 (m, 1H), 10.64 (br, s, 1H).

#### Linear Peptide, PL-49

Synthesis was carried out on 0.061 mmol scale. The purity of crude product was 64% (C_18_ RP-HPLC) and the yield after purification was 10.6 mg (20%, >98% purity) of white powder. RP-HPLC analysis, R_t_ = 13.5 (5-95% B in 30 min). MS (MALDI-TOF) m/z = 860.46 [M+H]^+^.

#### Linear Peptide, PL-47

Synthesis was carried out on a 0.048 mmol scale. The purity of the crude product was 63% (C_18_ RP-HPLC), and the yield after purification was 7.2 mg (20%, 98% purity) of white powder. RP-HPLC analysis, R_t_ = 9.5 (5–95% B in 30 min). MS (MALDI-TOF) m/z = 764.38 [M+H]^+^.

#### Linear Peptide, PL-48

Synthesis was carried out on a 0.048 mmol scale. The purity of the crude product was 79% (C_18_ RP-HPLC), and the yield after purification was 9.7 mg (25%, 98% purity) of white powder. RP-HPLC analysis, R_t_ = 14.4 (5–95% B in 30 min). MS (MALDI-TOF) m/z = 812.97 [M]^+^.

#### Linear Peptide, PL-52

Synthesis was carried out on a 0.061 mmol scale. The purity of the crude product was 79% (C_18_ RP-HPLC), and the yield after purification was 14.0 mg (25%, 97% purity) of white powder. RP-HPLC analysis, R_t_ = 15.5 (5–95% B in 30 min). MS (MALDI-TOF) m/z = 932.30 [M+H]^+^.

#### Linear Peptide, PL-55

Synthesis was carried out on a 0.041 mmol scale. The purity of the crude product was 73% (C_18_ RP-HPLC), and the yield after purification was 8.9 mg (23%, >98% purity) of white powder. RP-HPLC analysis, R_t_ = 18.4 (5–95% B in 30 min). MS (MALDI-TOF) m/z = 940.47 [M+H]^+^.

#### Linear Peptide, PL-56

Synthesis was carried out on a 0.041 mmol scale. The purity of the crude product was 60% (C_18_ RP-HPLC), and the yield after purification was 7.1 mg (19%, 98% purity) of white powder. RP-HPLC analysis, R_t_ = 20.1 (5–95% B in 30 min). MS (MALDI-TOF) m/z = 930.49 [M+H]^+^.

#### Linear Peptide, PL-57

Synthesis was carried out on a 0.041 mmol scale. The purity of the crude product was 75% (C_18_ RP-HPLC), and the yield after purification was 8.6 mg (24%, 98% purity) of white powder. RP-HPLC analysis, R_t_ = 16.1 (5–95% B in 30 min). MS (MALDI-TOF) m/z = 888.44 [M+H]^+^.

#### Linear Peptide, PL-74

Synthesis was carried out on a 0.041 mmol scale. The purity of the crude product was 63% (C_18_ RP-HPLC), and the yield after purification was 8.5 mg (20%, >98% purity) of white powder. RP-HPLC analysis, R_t_ = 19.3 (5-95% B in 30 min). MS (MALDI-TOF) m/z = 1043.62 [M+H]^+^.

#### Linear Peptide, PL-75

Synthesis was carried out on a 0.041 mmol scale. The purity of the crude product was 75% (C_18_ RP-HPLC), and the yield after purification was 9.7 mg (24%, 97% purity) of white powder. RP-HPLC analysis, R_t_ = 16.2 (5–95% B in 30 min). MS (MALDI-TOF) m/z = 1001.55 [M+H]^+^.

#### Linear Peptide, PL-64

Synthesis was carried out on a 0.061 mmol scale. The purity of the crude product was 76% (C_18_ RP-HPLC), and the yield after purification was 15.4 mg (24%, 98% purity) of white powder. RP-HPLC analysis, R_t_ = 17.2 (5–95% B in 30 min). MS (MALDI-TOF) m/z = 1056.95 [M+4]^+^.

#### Linear Peptide, PL-81

Synthesis was carried out on a 0.061 mmol scale. The purity of the crude product was 79% (C_18_ RP-HPLC), and the yield after purification was 16.3 mg (25%, 97% purity) of white powder. RP-HPLC analysis, R_t_ = 18.2 (5-95% B in 30 min). MS (MALDI-TOF) m/z = 1085.88 [M+4]^+^.

#### Linear Peptide, PL-100

Synthesis was carried out on a 0.061 mmol scale. The purity of the crude product was 52% (C_18_ RP-HPLC), and the yield after purification was 10.9 mg (16%, 97% purity) of white powder. RP-HPLC analysis, R_t_ = 17.0 (5-95% B in 30 min). MS (MALDI-TOF) m/z = 1114.55 [M+4]^+^.

#### Linear Peptide, PL-106

Synthesis was carried out on a 0.061 mmol scale. The purity of the crude product was 85% (C_18_ RP-HPLC), and the yield after purification was 15.8 mg (27%, 98% purity) of white powder. RP-HPLC analysis, R_t_ = 16.6 (5–95% B in 30 min). MS (MALDI-TOF) m/z = 972.58 [M+5]^+^.

### ELISA-based PBD-binding inhibition assay

 A biotinylated p-T78 (DPPLHSpTAIYADEE-NH_2_) peptide was diluted with coating solution (KPL, Inc., Gaithersburg, MD) to the final concentration of 0.3 µM, and then 50 µL of the resulting solution was immobilized onto a 96-well, streptavidin-coated plate (Nalgene Nunc, Rochester, NY). The wells were washed once with PBS + 0.05% Tween 20 (PBST), and incubated with 200 µl of PBS + 1% BSA (blocking buffer) for 1 h to prevent the unoccupied sites. Mitotic 293A lysates expressing HA-EGFP-Plk1 were prepared in the TBSN buffer (60 µg total lysates in 100 µL), applied onto the biotinylated peptide-coated ELISA wells immediately after mixing with the indicated amount of the competitor peptides, and then incubated with constant rocking for 1 h at 25 °C. Next, the plates were washed four times with PBST, and to detect the bound HA-EGFP-Plk1, the plates were incubated for 2 h with 100 µL/well of anti-HA antibody at a concentration of 0.5 µg/mL in the blocking buffer, and then the plates were washed five times. Furthermore, the plates were incubated with 100 µL/well of secondary antibody at a 1:1,000 dilution in the blocking buffer. Afterward, the plates were washed five times with PBST and incubated with 100 µL/well of 3,3′,5,5′-tetramethylbenzidine (TMB) substrate solution (Sigma, St. Louis, MO) until a desired absorbance was achieved. The reactions were terminated by the addition of 1 N H_2_SO_4_, and the optical densities were measured at 450 nm using an ELISA plate reader (Molecular Device, Sunnyvale, CA). Data are shown in Figures 2B, 4B, and 6B.

### Fluorescence polarization assays

Fluorescein-labeled peptides (5-carboxyfluorescein-DPPLHSpTAI-OH, final concentration: 25 nM) were incubated with the PBDs of Plk1, Plk2, and Plk3, respectively (final concentrations of buffer components: 10 mM Tris [pH 8.0], 1 mM EDTA, 50 mM NaCl, and 0.01% Nonidet P-40). Fluorescence polarization was analyzed 10 min after mixing all components in the 384-well format using a Molecular Devides SpectraMax Paradigm Multi-Mode Microplate Detection Platform. All experiments were performed in triplicate.

### X-ray crystallography

#### Protein purification and crystallization

The Plk1 PBD protein (residues 371−603) was purified as previously described [10]. Crystals were grown using the handing drop vapor diffusion method. The PBD protein at 12 mg/mL in a mixture of 10 mM Tris (pH 8), 0.5 M NaCl, 10 mM DTT, and 2% DMSO, and 2 mM of peptides were mixed with an equal volume of reservoir solution consisting of 31% (w/v) PEG 3350, 0.1 M bis-Tris (pH 5.5), and 240 mM MgCl_2_. Crystals began appearing overnight and reached maximum size after several days. Crystallographic and refinement statistics are summarized in Table 1. All figures were produced using the program PyMOL [19].

#### Accession Codes

Coordinates and structure factors have been deposited in the Protein Data Bank. PDB accession code: Plk1-PBD complex with PL-42; 4HY2, Plk1-PBD complex with PL-49; 4HAB, Plk1-PBD complex with PL-74; 4LKM, Plk1-PBD complex with PL-55; 4LKL.
